# Withaferin A Induces Proteasome-Dependent Degradation of Breast Cancer Susceptibility Gene 1 and Heat Shock Factor 1 Proteins in Breast Cancer Cells

**DOI:** 10.5402/2012/707586

**Published:** 2012-09-01

**Authors:** Xuan Zhang, Barbara Timmermann, Abbas K. Samadi, Mark S. Cohen

**Affiliations:** ^1^Department of Surgery, University of Kansas School of Medicine, Kansas City, KS 66160, USA; ^2^Department of Medicinal Chemistry, The University of Kansas, Lawrence, KS 66045, USA

## Abstract

The purpose of this study was to examine the regulation of prosurvival factors heat shock factor 1 (HSF1) and breast cancer susceptibility gene 1 (BRCA1) by a natural withanolide withaferin A (WA) in triple negative breast cancer cell lines MDA-MB-231 and BT20. Western analysis was used to examine alternations in HSF1 and BRCA1 protein levels following WA treatment. A protein synthesis inhibitor cycloheximide and a proteasome inhibitor MG132 were used to investigate the mechanisms of HSF1 and BRCA1 regulation by WA. It was found that WA induced a dose-dependent decrease in HSF1 and BRCA1 protein levels. Further analysis showed that levels of HSF1 and BRCA1 proteins decreased rapidly after WA treatment, and this was attributed to WA-induced denaturation of HSF1 and BRCA1 proteins and subsequent degradation via proteasome-dependent, and protein-synthesis dependent mechanism. In summary, WA induces denaturation and proteasomal degradation of HSF1 and BRCA1 proteins. Further studies are warranted to examine the contribution of HSF1 and BRCA1 depletion to the anticancer effects of WA in breast cancer.

## 1. Introduction

Withaferin A (WA) is a steroidal lactone naturally occurring in the medicinal plant *Withania somnifera*, which has recently been found to have potent anticancer and radio-sensitizing effects in human cancer cell lines and in animal cancer models without any noticeable systemic toxicity [1]. For breast cancer, WA has been found to induce cell cycle arrest and apoptosis in vitro and inhibit tumor growth in mouse models with xenograft of human breast cancer cells [2, 3]. The mechanism of action for WA is currently under extensive investigation. A number of targets have been identified, including nuclear factor-*κ*B [4], Notch [5], Hsp90 [6], STAT3 [7], and vimentin [8]. 

 We hypothesized that WA may target breast cancer susceptibility gene-1 (BRCA1) and heat shock factor 1 (HSF1) in breast cancer cells. BRCA1 plays a critical role in DNA double-strand break repair. Although BRCA1 mutation is linked to increased risk of breast cancer [9] and nonphysiological overexpression of BRCA1 induces apoptosis [10], recent research showed that reduced BRCA1 expression resulted in decreased viability of MDA-MB-231 cells [11], suggesting functional BRCA1 as a therapeutic target. It has also been reported that turnover of BRCA1 is involved in radiation-induced apoptosis [12]. About 89% of triple-negative breast cancers (defined by deficiency of estrogen-receptor, progesterone receptor, and HER-2 receptor) have wild-type BRCA1 [13], as such, BRCA1 may serve as a therapeutic target for most cases of this difficult breast cancer subtype. Another potential target is Heat shock factor 1 (HSF1), which is a transcription factor that stimulates synthesis of heat shock proteins and thus a promising target for cancer therapy. HSF1 has recently been shown as a facilitator of transformation in breast cancer [14, 15]. Inhibition of HSF1 suppresses the progression of a wide spectrum of cancers [16, 17]. 

In the present study, we intended to examine the effects of WA on BRCA1 and HSF1 protein expression in MDA-MB-231 and BT20 triple-negative breast cancer cells as potential mechanisms of its anticancer action. MDA-MB-231 and BT20 cells are both triple-negative human breast cancer cell lines with functional BRCA1. MDA-MB-231 cells are basal B adenocarcinoma, and BT20 cells are basal A invasive ductal carcinoma.

## 2. Materials and Methods

### 2.1. Cell Lines and Reagents

Triple negative MDA-MB-231 and BT20 human breast cancer cell lines were purchased from the American Type Culture Collection (ATCC; Manassas, VA) and cultured in DMEM containing 5% fetal bovine serum (FBS), 100 units/Ml penicillin, 100 *μ*g/mL streptomycin, and 2 mM glutamine. WA was purchased from ChromaDex (Irvine, CA). The antibody against HSF1 was purchased from Cell Signaling Technology (Danvers, MA) and the antibody against BRCA1 was purchased from EMD4Biosciences (Darmstadt, Germany). The anti-*β*-actin antibody was purchased from Millipore (Billerica, MA). The anti-Annexin V-FITC-conjugated and propidium iodide (PI) were purchased from BD Bioscience (Rockville, MD). 

### 2.2. Annexin V-FITC/PI Flow Cytometry

The apoptotic effects of WA were examined using Annexin V-FITC/PI flow cytometry as previously described [18]. Briefly, MDA-MB-231 or BT20 cells were treated with increasing doses of WA for 24 h. Detached and adherent cells were then collected and labeled for 15 min at room temperature with annexin V- FITC (1 *μ*g/mL) and with PI (40 *μ*g/mL) and immediately analyzed on a BD LSRII flow cytometer (BD Biosciences) using BD FACSDiva6.0 software. 

### 2.3. Western Analysis

Total proteins were extracted using radioimmuno-precipitation assay buffer (20 mM Tris (pH 7.5), 150 mM NaCl, 1% IGEPAL CA-630, 0.5% sodium deoxycholate, 1 mM ethylenediaminetetra-acetic acid, and 0.1% SDS) containing a protease/phosphatase inhibitor cocktail (0.1 mg/mL PMSF, 30 *μ*L/mL of aprotinin, 5 *μ*g/mL of leupeptin, and 1 mM sodium orthovanadate; Sigma). Protein concentrations were determined using the BCA protein assay reagent kit (Pierce, Rockford, IL). Equal amount of proteins were subjected to SDS-polyacrylamide gel electrophoresis (PAGE) and electroblotted onto nitrocellulose membranes (Hybond; Amersham, Piscataway, NJ). After blocking with 3% nonfat dry milk in PBS for 1 h at room temperature, blots were incubated with appropriate primary antibodies overnight at 4°C. Blots were then washed and incubated with appropriate horseradish peroxidase (HRP)-conjugated secondary antibodies for 1 h, and proteins of interests were detected using a chemiluminescence kit (Thermo Scientific, Rockford, IL). Next, blots were stripped and reprobed for *β*-actin. The expression level of each protein was normalized to the level of *β*-actin. 

To examine whether WA modulates HSF1 and BRCA1 protein stability, MDA-MB-231 cells were treated with 100 *μ*g/mL of protein synthesis inhibitor cycloheximide (CHX) in the absence or presence of 2.5 *μ*M WA for 6 or 24 h. Cells were then collected and proteins in triton-soluble or triton-insoluble fractions were isolated as described previously [19], and subjected to Western analysis for HSF1, BRCA1 and *β*-actin. 

To examine whether downregulation of HSF1 and BRCA1 protein levels was attributed to proteasome-dependent protein degradation, MDA-MB-231 cells were pretreated with 30 *μ*M of proteasome inhibitor MG132 for 1 h before treatment with 2.5 *μ*M WA for 6 h. Cells were then collected and proteins in triton-soluble or triton-insoluble fractions were isolated and subjected to Western analysis.

### 2.4. Statistical Analysis

All data were analyzed using SPSS Version 17.0 software (SPSS, Inc.). ANOVA was used for comparison across treatment regimes. When an *F* test indicated statistical significance, post hoc analysis was made using the Tukey's honestly significant difference procedure. Significance was set at *P* < 0.05 for all comparisons.

## 3. Results

### 3.1. BT20 Cells Are More Sensitive to the Apoptotic Effect of WA than MDA-MB-231 Cells

**Figure 1:**
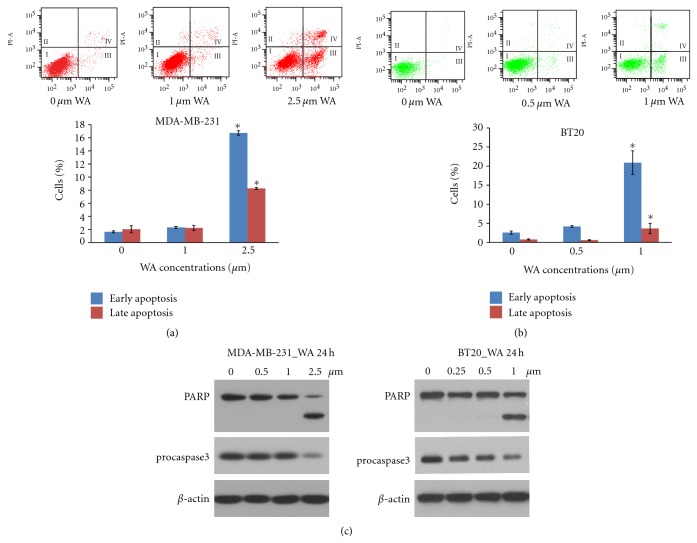
A and B: Effects of WA on MDA-MB-231 (a) and BT20 (b) cell apoptosis as measured by Annexin V-FITC-propidium iodide flow cytometry. Cells were treated with indicated concentrations of WA for 24 h. Results were presented as mean (*n* = 3) SD. ∗, *P* < 0.05, significantly different from control by one-way ANOVA. (C) Western analysis of the expression of PARP and caspase-3 in MDA-MB-231 and BT20 cells. Cells were treated with indicated concentrations of WA for 24 h. Western for *β*-actin was conducted to confirm equal loading of proteins.

Although the apoptotic effect of WA in MDA-MB-231 cells has been reported before, its effects in other triple-negative cell lines are not known. Considering the heterogeneous nature of the triple-negative breast cancer subtype, we decided to include both MDA-MB-231 and BT20 cells in this study. We first conducted Annexin V-FITC/PI flow cytometry to examine the apoptotic effects of WA. 2.5 *μ*M of WA induced significant increase in apoptosis in MDA-MB-231 cells at 24 h (Figure 1(a)). In BT20 cells, 1 *μ*M of WA induced a significant increase in apoptosis at 24 h (Figure 1(b)), indicating higher sensitivity of BT20 to the apoptotic effects of WA. 

The induction of apoptosis by WA in MDA-MB-231 and BT20 cells at 24 h was accompanied by increased poly (ADP-ribose) polymerase (PARP) cleavage and caspase-3 activation, as shown by Western analysis (Figure 1(c)). 

### 3.2. WA Markedly Downregulates HSF1 and BRCA1 Proteins in MDA-MB-231 and BT20 Cells

**Figure 2:**
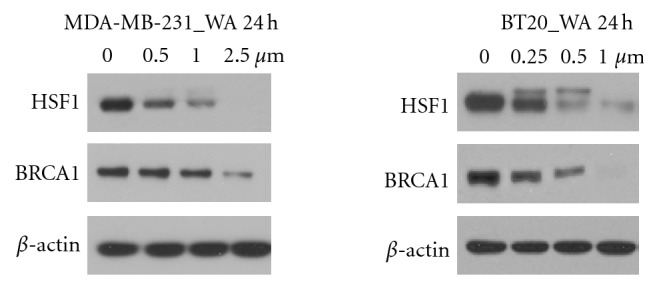
A. Western analysis of the expression of HSF1 and BRCA1 proteins in MDA-MB-231 and BT20 cells. Cells were treated with indicated concentrations of WA for 24 h.

The regulation of two prosurvival proteins HSF1 and BRCA1 in MDA-MB-231 and BT20 cells by WA was examined using Western analysis. Both HSF1 and BRCA1 proteins were found to be markedly down-regulated by WA in a dose-dependent manner (Figure 2). In MDA-MB-231 cells, HSF1 and BRCA1 protein levels diminished after 2.5 *μ*M WA treatment for 24 h. In BT20 cells, these two proteins diminished after 1 *μ*M WA treatment for 24 h. 

### 3.3. Downregulation of HSF1 and BRCA1 is Predominantly due to Decreased Protein Stability

**Figure 3:**
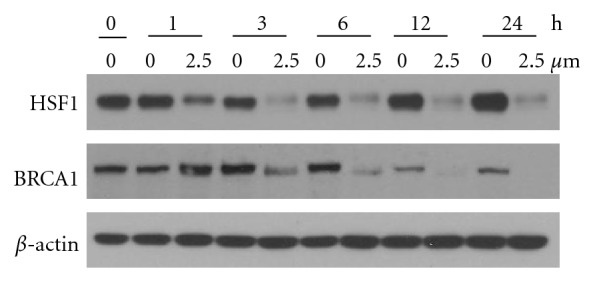
Western analysis of the time course the effects of WA on HSF1 and BRCA1 proteins levels in MDA-MB-231 cells. Cells were treated with 0.125% DMSO or 2.5 *μ*M WA for 0, 1, 3, 6, or 24 h.

To examine the time course of WA-induced downregulation of HSF1 and BRCA1, MDA-MB-231 cells were treated with vehicle (0.125% DMSO) or 2.5 *μ*M WA for 0, 1, 3, 6, 12, and 24 h. Western analysis for HSF1 and BRCA1 showed that both HSF1 and BRCA1 proteins were rapidly decreased following WA treatment. HSF1 protein levels were decreased at as early as 1 h post treatment, and BRCA1 protein levels were decreased starting at 3 h post treatment (Figure 3), as compared to vehicle groups. We also noticed that BRCA1 protein levels in vehicle groups vary across the time points, with its levels at 12 h and 24 h much lower than those at 3 h and 6 h after treatment. 

**Figure 4:**
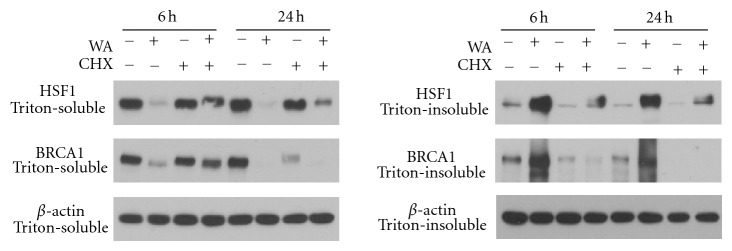
Effects of protein synthesis inhibitor CHX (100 *μ*g/mL) alone or in combination with WA (2.5 *μ*M) on HSF1 and BRCA1 protein expression in Triton-soluble and Triton-insoluble fractions of MDA-MB-231 cell lyses at 6 h and 24 h.

We then used a protein synthesis inhibitor CHX to examine if WA is able to decrease HSF1 and BRCA1 protein stability. MDA-MB-231 cells were treated with CHX alone or in combination with 2.5 *μ*M WA for 6 or 24 h. We found that co-treatment with CHX + WA markedly decreased the protein levels of HSF1 and BRCA1 at both 6 h and 24 h, as compared to CHX alone in Triton-soluble fractions, suggesting that WA may downregulate HSF1 and BRCA1 protein levels posttranslationally by decreasing their stability (Figure 4). As expected, WA treatment alone caused a decrease in HSF1 and BRCA1 protein levels in Triton-soluble fractions. Meanwhile, their levels in Triton-insoluble fractions were increased by WA. Co-treatment with CHX partially blocked WA-induced decrease in levels of HSF1 and BRCA1 proteins in Triton-soluble fractions.

### 3.4. WA Induces Proteasome-Dependent Degradation of HSF1 and BRCA1 Proteins

**Figure 5:**
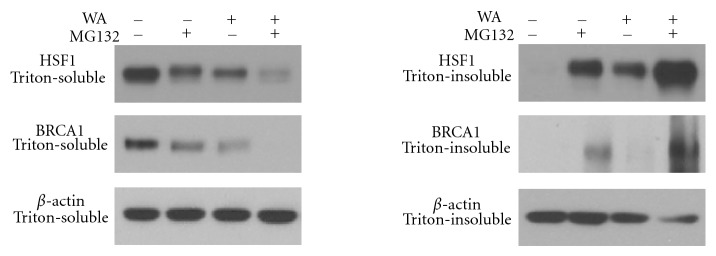
Western analysis of the effects of a proteasome inhibitor MG132 (30 *μ*M) alone or in combination with WA (2.5 *μ*M) on HSF1 and BRCA1 protein expression in Triton-soluble and Triton-insoluble fractions of MDA-MB-231 cell lyses at 6 h post WA treatment.

To examine if downregulation of HSF1 and BRCA1 protein levels by WA is mediated by proteasomal protein degradation, MDA-MB-231 cells were pretreated with 30 *μ*M of a proteasome inhibitor MG132 for 1 h before treatment with 2.5 *μ*M WA for 6 h (Figure 5). WA alone decreased the protein levels of HSF1 and BRCA1 in Triton-soluble fractions and increased their levels in Triton-insoluble fractions. Pretreatment with MG132 before WA further increased the levels of HSF1 and BRCA1 proteins in Triton-insoluble fractions as compared to either MG132 or WA alone, suggesting that WA induced denaturation and proteasome-dependent degradation of HSF1 and BRCA1 proteins. 

 We also observed that MG132 caused a decrease in HSF1 and BRCA1 protein levels in Triton-soluble fractions while increasing its levels in Triton-insoluble fractions, as compared to controls. 

## 4. Discussion 

In the present study, we demonstrated that (1) the natural withanolide WA has proapoptotic effects in both MDA-MB-231 (basal B, adenocarcinoma) and BT20 (basal A, invasive ductal carcinoma) triple-negative breast cancer cells, with BT20 cells showing higher sensitivity; (2) the apoptotic effects of WA are associated with diminished levels of HSF1 and BRCA1 proteins; (3) rapid downregulation of HSF1 and BRCA1 proteins by WA is predominantly attributed to post-translational modulation via protein denaturation and proteasomal degradation; (4) WA-induced down-regulation of HSF1 and BRCA1 proteins is protein-synthesis dependent. 

We found that downregulation of HSF1 and BRCA1 proteins occurred rapidly following WA treatment, suggesting potential decrease in protein stability. This was confirmed by co-treatment with a protein synthesis inhibitor CHX along with WA, as HSF1 and BRCA1 levels were much lower in CHX + WA group as compared to CHX treatment alone in Triton-soluble fractions. It is worth mentioning that WA-induced downregulation of HSF1 and BRCA1 in Triton-soluble fractions was partially/temporarily blocked by CHX, suggesting that *de novo* protein synthesis is needed in this process. One possible explanation is that certain labile or short-lived proteins are involved in WA-induced degradation of HSF1 and BRCA1 proteins. 

Experiments using a proteasome inhibitor MG132 demonstrated that WA caused HSF1 and BRCA1 protein denaturation and proteasome-dependent degradation, as levels of these proteins in Triton-insoluble fractions were much higher in MG132 + WA group as compared to either MG132 or WA alone. The involvement of the ubiquitin-proteasome pathway also suggests that inhibition of WA-induced HSF1 and BRCA1 protein downregulation by CHX could be due to CHX-induced depletion of short-lived E3 ubiquitin ligases, which warrants further investigation. 

It is intriguing that MG132 alone induced a decrease in both HSF1 and BRCA1 protein levels in Triton-soluble fractions while inducing an increase in their levels in Triton-insoluble fractions of breast cancer cell protein extract. We speculate that this alteration may result from MG132-induced accumulation of HSF1 and BRCA1 into nuclear granules, rendering it insoluble. It has been reported that proteasome inhibition by MG132 or lactacystin could cause conformational changes of HSF1 molecules and trigger their accumulation into nuclear granules [20]. Although it is not known whether MG132 can cause similar subnuclear compartmentation of BRCA1 protein, Motoaki Sano demonstrated that MG132 treatment induced translocation of subnuclear compartment shift of another transcription factor peroxisome proliferator-activated receptor coactivator-1 (PGC-1) rendering it insoluble [21]. 

 Additionally, we noticed that BRCA1 protein levels in vehicle groups vary across the time points, with its levels at 12 h and 24 h much lower than those at 3 h and 6 h after treatment. Earlier research has demonstrated that levels of BRCA1 protein expression fluctuate during cell cycle [22]. BRCA1 has been shown to interact with both DNA and cellular proteins [23]. The regulation of BRCA1 protein expression is likely complicated. 

As mentioned earlier, BRCA1 has been found to be critically involved in cell survival in MDA-MB-231 cells [11]. BRCA1 also protects cancer cells against oxidative stress by regulating antioxidant responses [24]. HSF1, a stress response protein and an activator of heat-shock protein encoding genes, is implicated in breast cancer initiation and progression [25]. Increased HSF1 is associated with reduced breast cancer survival [26]. The fact that attenuated expression of HSF1 and BRCA1 proteins were associated with the apoptotic effects of WA in both MDA-MB-231 and BT20 cells suggests that they may play a role in the anticancer action of WA in triple negative breast cancer cell lines. Cells may not respond to DNA damage caused by WA when these prosurvival factors are diminished, and thus enter programmed cell death. However, directly confirming their roles in the proapoptotic effect of WA could be challenging. Because apoptotic concentrations of WA almost completely depleted BRCA1 and HSF1 proteins at posttranslational level by protein denaturation, overexpression of BRCA1 or HSF1 protein may not be feasible. 

In summary, we report here that WA causes denaturation and proteasome-dependent degradation of HSF1 and BRCA1 proteins in MDA-MB-231 and BT20 triple-negative breast cancer cells. Further studies are needed to examine the contribution of HSF1 and BRCA1 depletion to the anticancer effects of WA in breast cancer.

## Abbreviations

HSF1: Heat shock factor 1
BRCA1: Breast cancer susceptibility gene 1
CHX: Cychloheximide.
